# The Use of Artificial Intelligence for the Classification of Craniofacial Deformities

**DOI:** 10.3390/jcm12227082

**Published:** 2023-11-14

**Authors:** Reinald Kuehle, Friedemann Ringwald, Frederic Bouffleur, Niclas Hagen, Matthias Schaufelberger, Werner Nahm, Jürgen Hoffmann, Christian Freudlsperger, Michael Engel, Urs Eisenmann

**Affiliations:** 1 Department of Oral and Maxillofacial Surgery, University of Heidelberg, 69120 Heidelberg, Germany; frederic.bouffleur@med.uni-heidelberg.de (F.B.); juergen.hoffmann@med.uni-heidelberg.de (J.H.); christian.freudlsperger@med.uni-heidelberg.de (C.F.);; 2 Institute of Medical Informatics, University of Heidelberg, 69120 Heidelberg, Germany; friedemann.ringwald@med.uni-heidelberg.de (F.R.);; 3 Institute of Biomedical Engineering, Karlsruhe Institute for Technology, 76131 Karlsruhe, Germany

**Keywords:** artificial intelligence, deep learning, craniosynostoses, photogrammetry, congenital abnormalities, trigonocephaly

## Abstract

Positional cranial deformities are a common finding in toddlers, yet differentiation from craniosynostosis can be challenging. The aim of this study was to train convolutional neural networks (CNNs) to classify craniofacial deformities based on 2D images generated using photogrammetry as a radiation-free imaging technique. A total of 487 patients with photogrammetry scans were included in this retrospective cohort study: children with craniosynostosis (n = 227), positional deformities (n = 206), and healthy children (n = 54). Three two-dimensional images were extracted from each photogrammetry scan. The datasets were divided into training, validation, and test sets. During the training, fine-tuned ResNet-152s were utilized. The performance was quantified using tenfold cross-validation. For the detection of craniosynostosis, sensitivity was at 0.94 with a specificity of 0.85. Regarding the differentiation of the five existing classes (trigonocephaly, scaphocephaly, positional plagiocephaly left, positional plagiocephaly right, and healthy), sensitivity ranged from 0.45 (positional plagiocephaly left) to 0.95 (scaphocephaly) and specificity ranged from 0.87 (positional plagiocephaly right) to 0.97 (scaphocephaly). We present a CNN-based approach to classify craniofacial deformities on two-dimensional images with promising results. A larger dataset would be required to identify rarer forms of craniosynostosis as well. The chosen 2D approach enables future applications for digital cameras or smartphones.

## 1. Introduction

Skull deformities manifest in approximately 16% [1,2] of newborns, with craniosynostosis diagnosed at a notably lower incidence of 0.05%. This condition entails the premature ossification of one of the five skull sutures, impeding the perpendicular growth of the neurocranium along the affected suture [3,4]. Isolated craniosynostosis exhibits a multifactorial etiology, though familial aggregation is noted in certain cases. Syndromic craniosynostosis, affecting multiple sutures, is commonly associated with craniofacial syndromes like Morbus Pfeiffer, M. Apert, M. Crouzon, or M. Muenke, often linked to mutations in the Fibroblast Growth Factor Receptor gene (FGFR) [5].

Craniosynostoses hinder regular neurocranial growth, leading to craniofacial deformities with significant psychosocial implications and an elevated risk of intracranial pressure elevation or neuropsychological development deficits [6,7]. The early detection of pathological growth patterns is imperative, often discernible through specific clinical findings and growth patterns.

Patients with craniofacial deformities typically present in early infancy and are conventionally diagnosed through clinical examination, ultrasound, or imaging modalities like Computed Tomography (CT) or Magnetic Resonance Imaging (MRI) [8,9]. However, the use of tomographic techniques, especially in young patients, necessitates general anesthesia and carries the risk of radiation exposure in CT imaging, a concern given the increased susceptibility to malignancies during early development [10,11]. Still, the advantage of incidental relevant findings in CT or MRI imaging and the confirmatory function of imaging need to be considered and set into relation.

Three-dimensional (3D) photography emerges as a non-invasive alternative for skull growth analysis [12,13,14]., demonstrating independence from examiner-related biases [15,16,17] and possible subjective bias [18,19,20]. Traditional methods, such as CT scans with manual cephalometric measurements, may not be mandatory for single-suture synostosis diagnosis, as suggested by several authors to date [21,22,23]. Photogrammetry, or any form of optical 3D scanning, efficiently captures craniosynostosis morphology, aiding in cephalometric measurements and surgical result quantification [14,16,23,24]. Nevertheless, the use of a 3D scan as a diagnostical tool needs an experienced surgeon for interpretation. This study proposes a theoretical basis for a diagnostic approach combining clinical examination with an AI-based classification of a 3D photo face scan for single-suture synostosis diagnosis [6,12,13].

To this day, many craniofacial centers still rely on CT scans for the diagnostic assessment of cranial deformity [25,26], and primary or secondary medical care units often lack the expertise to distinguish positional deformities from craniosynostosis with certainty. Recognizing the efficacy of convolutional neural networks (CNNs) and deep learning (DL) in medical image analysis, particularly in radiology [27,28], we aim to leverage these technologies for classifying pathological growth patterns in craniosynostosis. While CNNs have excelled in 2D image classification, such as 2D X-rays, pathohistological scans or photos of skin cancer [29,30,31], their application to 3D data, such as face scans, is limited by the scarcity of the relevant literature and pre-trained neural networks [30]. However, when transitioning to 3D images like CT or MRI scans, the challenges multiply due to increased data complexity and computational demands.

The representation of 3D images either through polygon models or native 3D imaging demands a more intricate understanding and processing. Neural networks tailored for 3D data, like 3D CNNs, need to be employed. These models are computationally intensive and require significantly more resources for training and inference, alongside a large annotated dataset, which are often scarce in the medical domain, especially for rare conditions such as craniosynostosis. The geometric and topological intricacies in 3D medical images, such as surface scans depicted by polygon models, necessitate advanced preprocessing and augmentation techniques to ensure accurate representation and subsequent classification.

Furthermore, the heterogeneity in medical imaging data, stemming from different imaging modalities, varying acquisition protocols or scanning hardware, and patient-specific variations, adds an additional layer of complexity. Ensuring robustness and generalizability across diverse medical imaging datasets is crucial, which requires meticulous dataset curating. With the limited data on craniosynostosis in a single tertiary care center, focusing on 2D data is more viable as they require fewer computational resources and are easier to manage than 3D data, enabling quicker iterations and potentially more accurate predictions with the available data.

Our project aims to establish the technological foundation for a diagnostic tool using 2D images extracted from patient-specific photogrammetric surface scans. This tool seeks to enhance diagnostics in primary care and diminish reliance on CT scans for infantile patients. Unlike approaches requiring extensive manual preprocessing, our straightforward 2D approach, derived from photogrammetric scans, may be applicable independently of 3D scanning devices, utilizing standard digital cameras or smartphones to support pediatricians and the primary medical sector.

This study addresses two key questions:

Can neural networks reliably differentiate craniosynostosis from non-synostotic patients?
Can neural networks reliably classify different types of common craniofacial deformities?

## 2. Materials and Methods

### 2.1. Study Design and Ethics

The study protocol was reviewed and approved by the Ethics Committee of the Medical Faculty of the University of Heidelberg (ethics number S-237/2009). The study was carried out according to the Declaration of Helsinki and written informed consent was obtained from parents.

### 2.2. Patients

The database of the Department of Oral and Cranio-Maxillofacial Surgery at the University Hospital of Heidelberg underwent a query to identify children with cranial deformities spanning the years 2011 to 2020. Inclusion criteria comprised patients aged 0–14 months, who obtained parental consent and underwent a routine photogrammetric scan at our department for head form evaluation. Validation of the initial clinical diagnosis was contingent upon specialist opinion, surgical intervention, additional diagnostics, or subsequent scans. Enrolled patients exhibited craniofacial deformities, posterior positional deformities, or sagittal/metopic synostosis. Exclusion criteria encompassed a history of complex craniofacial syndromic deformities, non-synostotic and non-positionally associated craniofacial deformities, as well as a history of prior craniofacial surgery. Due to the limited number of cases, lambdoid and coronary craniosynostoses (right, left, and bilateral) were excluded during data collection. Excluding small subgroups in training neural networks in general is essential to prevent model bias and unreliable generalization. Small subgroups lack the robustness necessary for the model to discern meaningful patterns, making it prone to overfitting and misinterpreting rare cases. Excluding the underrepresented groups in this collective ensures a focus on more prevalent and representative cases, fostering a model that better captures the nuances of common pediatric craniofacial structures and conditions. A total of 50 patients per group was set as the cutoff to be able to distribute patients properly between training, validation and test sets.

The healthy control group was recruited from children seen in our outpatient clinic who either did not have a deformity or had a positional deformity within the threshold of a normal range. Every patient with a 3D photogrammetry scan in our clinical routine is analyzed with the Cranioform Analytics Software 4.0 (Cranioform AG, Alpbach, Switzerland). To be qualified as a healthy control group patient, the threshhold was defined to be a cranial vault asymmetry index (CVAI) of <3.5% and a cranial index (CI) of <90. Furthermore, healthy twins and siblings of patients, routinely 3D scanned in our department, were also included. The collected dataset was manually screened to exclude other forms of craniomaxillofacial anomalies that could be confounding, such as cleft lip patients or other syndromic patients with relevant asymmetries.

### 2.3. Cephalometric Measurements and Analytics

**Figure 1:**
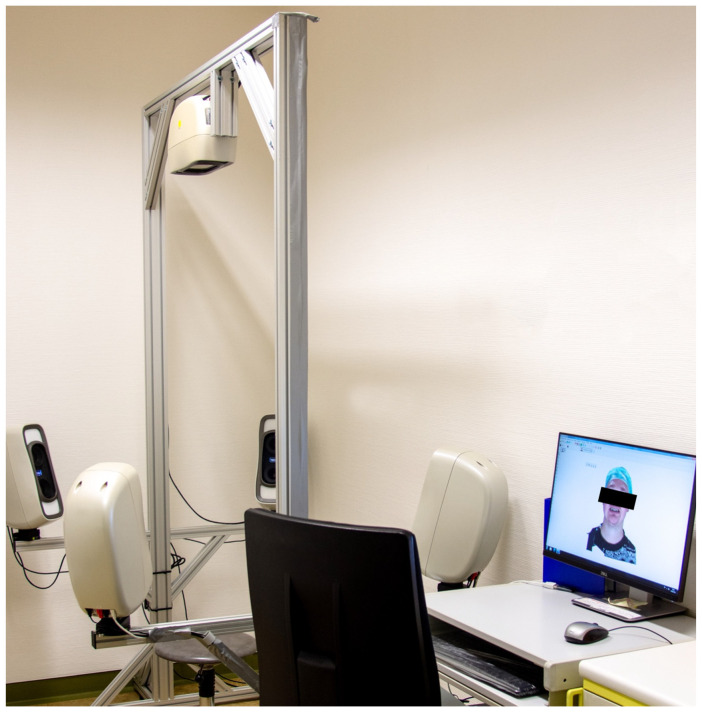
Vectra-M5 360 (Canfield Scientific Inc., Fairfield, NJ, USA) System is a three-dimensional stereophotogrammetry camera system that consists of five cameras.

The patient’s 3D surface model was recorded in the daily routine using a 3D X-ray-free photogrammetry system in the Department of Oral and Maxillofacial Surgery at the University of Heidelberg. All photographic scans (recording time: 1.5 ms) were performed using a stereophotogrammetric Canfield VECTRA-M5 360 system (Canfield Science, Fairfield, NJ, USA) using a standardized recording protocol (Figure 1).

**Figure 2:**
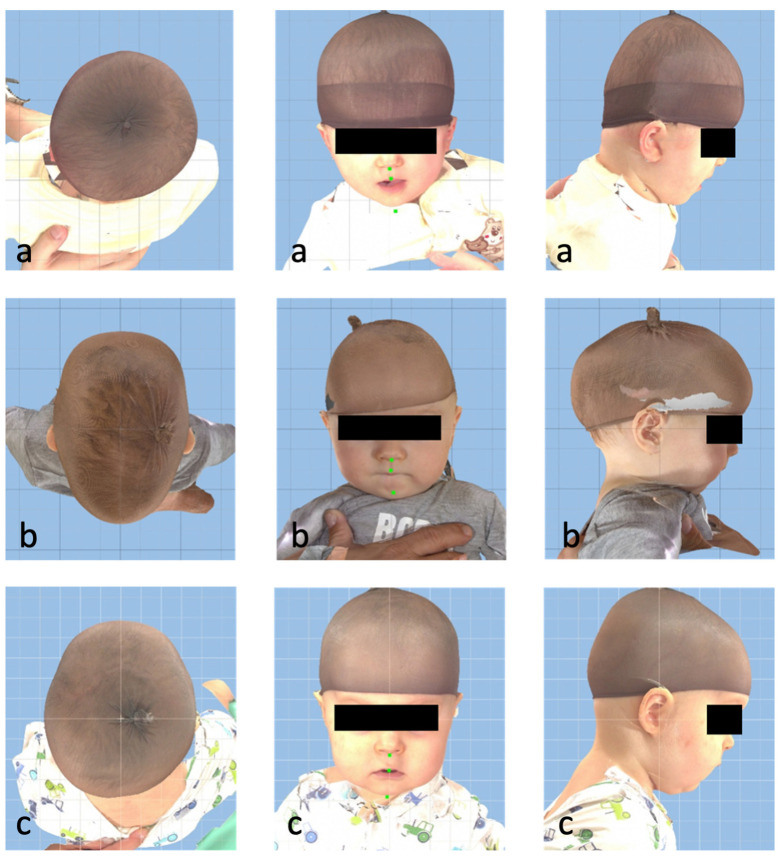
Three images per patient: frontal, lateral, and top-down ((**a**) positional plagiocephaly right side; (**b**) scaphocephaly; (**c**) healthy).

The system consists of 5 stereophotogrammetric cameras, allowing one to calculate a 3D surface with textures or 2D perspectives of the scanned patient. The infant was placed on the chair in the middle while the images were acquired. To properly show the head shape, a tight cap was placed on the head (Figure 2).

The data were orientated and spatially annotated using Cranioform Analytics 4.0 software (Cranioform, Alpnach, Switzerland), the Mirror software 2.0 (Canfield Science, Fairfield, NJ, USA), and Geomagic Studio 6.0 (Raindrop Inc., Durham, NC, USA). For the training, frontal, lateral, and top-down images of the patient’s head were exported.

### 2.4. Approach and Preprocessing

This study was based on 2D images (1600 × 1400 pixels, jpeg) calculated from the photogrammetric data. No advanced preprocessing (e.g., segmentation or ROI definition) was applied to the data. By using 2D images, this approach should be optimized for regular photos to be used in perspective without 3D scanning. As neuronal networks perform best on images of the same width and height, images were automatically rescaled to 1600 × 1600 and center-cropped.

Data augmentation was conducted using established methods (rotational, flip, crop, and color jitter) and pixels were normalized via mean image subtraction. Augmentation techniques were used from the PyTorch transforms library. The dataset of all annotated images was split into training (60%), validation (20%), and test data (20%). The test data set was held back and not used for training or validation so that we could validate the network afterward.

### 2.5. Network Architecture

Due to the limited amount of image data, training from scratch did not yield satisfactory results, and pre-trained networks from PyTorch were utilized. This greatly reduced the training effort as pre-trained networks can already distinguish basic image features like edges, color changes, etc. The loss function was equipped with individual weights to account for the strongly imbalanced class distribution of the five possible classification results.

**Figure 3:**
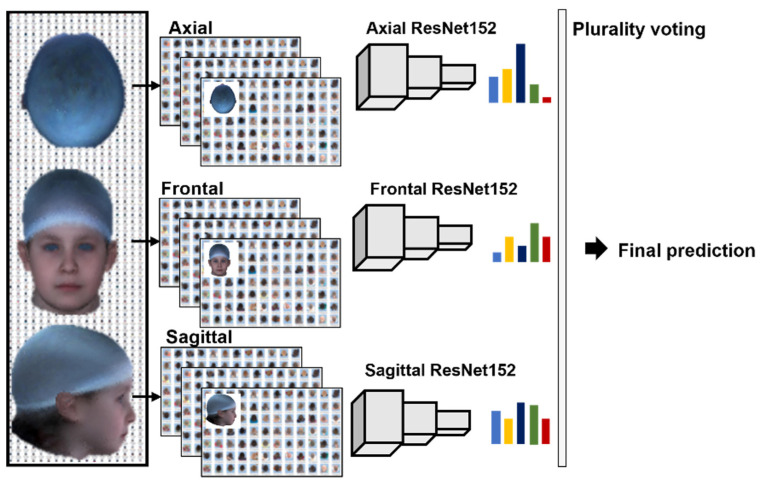
The architecture of the ResNet 152 3-Net approach. For each projection (axial, frontal, and sagittal) a ResNet152 delivers a classification. Plurality voting selects the classification with the highest probability between the three predictions to a final prediction for the cranial deformity (patient picture was artificially generated).

Since the different 2D images—en-face, top-down, and profile—contained different views and features of the head, one individual pre-trained CNN was used for each projection. With this approach, each individual network became an expert for the specific projection. During preliminary testing on the available data, the pre-trained ResNet152 (RN152) performed best compared to other models (ResNext, Alexnet, InceptionV3, and GoogLe-Net) and thus was chosen for further processing. The three individual RN152s for each projection were trained with the corresponding image data. Each net predicted the related images after completing the training, validation, and test loop. Before the final classification, the performance of each network was fine-tuned individually, optimizing loss by adapting the available hyperparameters. Resulting in three predictions, the final result was calculated via plurality voting by summing up the certainties of each classification network and selecting the class with the highest overall score as illustrated in the architecture in Figure 3.

### 2.6. Validation and Statistical Evaluation

In this study, the final classification results were derived from the independent test set, which was excluded from both training and validation phases, ensuring unbiased evaluation. To enhance reliability, tenfold cross-validation was employed, involving ten unique splits of the training and validation sets. This approach yielded an average performance for the test set, enhancing robustness and strengthening the validity of the results. Subsequently, global accuracy, precision, sensitivity, and specificity values were calculated for all patients as well as for each individual class.

### 2.7. Addressing the Research Questions

To answer the first main research question: “Can a combination of neural networks reliably indicate the necessity of surgery in patients with cranial deformities?”, the collective and the respective data were partitioned into two cohorts: Cohort 1 with patients with an indication for surgery and Cohort 2 with no indication for surgery (n = 260: healthy or positional posterior plagiocephaly on the right and left side).

To answer the second main research question: “Can a combination of neural networks reliably classify different types of craniosynostosis, position-induced deformities and healthy controls?”, the collective was partitioned into five groups (healthy, deformational posterior plagiocephaly left, deformational posterior plagiocephaly right, scaphocephaly, and trigonocephaly).

## 3. Results

### 3.1. Patient Collective

Following the application of inclusion criteria, 487 patients could be enrolled in this study. In total, 73% of the cohort were male (n = 356), while 27% (n = 131) were female. The categorized cohorts included healthy controls (n = 54), positional posterior plagiocephaly left (n = 72), positional posterior plagiocephaly right (n = 134), scaphocephaly (n = 138), and trigonocephaly (n = 89). There were n = 227 patients with an indication for surgery (craniosynostosis in the form of scaphocephaly or trigonocephaly) and n = 260 patients with no indication for surgery (healthy or positional posterior plagiocephaly on the right and left side).

### 3.2. Network Performance

The final results can be seen in Table 1 and Table 2. Table 1 corresponds to the differentiation of the group with an indication for surgery or healthy control; Table 2 shows the results of the five classes. The results for the first question (Can we determine the indication for surgery or not?) showed an accuracy for both classes of 90%. All other statistical evaluation parameters averaged over 85%, showing good classification potential for the underlying cohort.

In addressing the second research question, a tenfold cross-validation was conducted on the statistical measures, as illustrated in Table 2. It was generally observed that the classes with a higher number of patients (scaphocephaly, plagiocephaly right, and trigonocephaly) exhibited superior performance. With respect to sensitivity, the scaphocephaly group yielded the best results, while the plagiocephaly left group showed the least favorable outcome. Precision was highest in the scaphocephaly group and lowest in the healthy control group. A commendable level of accuracy, ranging from 84 to 96%, was achieved across all subgroups. However, the sensitivity for the healthy and plagiocephaly patients was notably lower, standing at 73% and ranging from 45 to 76%, respectively. Overall, the findings are encouraging, barring the subgroups healthy and plagiocephaly left, where the results were less satisfactory.

Table 3 presents the metrics of true positive, true negative, false positive, and false negative concerning the first research question related to the differentiation between the classes indicating surgery and no surgery requirement. Both the true positive and true negative rates exceed 90%, while the false positive and false negative rates are below 10%, showcasing a highly satisfactory performance.

Table 4 delineates the metrics, including true positive, true negative, false positive, and false negative, pertinent to the second research question addressing the differentiation among the five observed classes: healthy, plagiocephaly left, plagiocephaly right, scaphocephaly, and trigonocephaly. Despite the less favorable performance of true positives in the healthy class, it surpasses the randomness of a dice throw. Nevertheless, it is not on par with the performance observed in the other groups, underscoring the necessity for additional validation with a healthy control group.

## 4. Discussion

Convolutional neural networks are progressively used in the automated classification of medical images and represent a central tool in image-based machine learning. Critical for the performance of these methods are the amount of data, the quality of annotation, and the homogeneity of data distribution to the classes. To our knowledge, we present the largest collective of photogrammetry scans of craniofacial patients assessed using convolutional neural networks. The observed 3:1 male-to-female ratio aligns with the existing literature, particularly in the context of metopic and sagittal synostosis [32]; we, therefore, deem it a representable collective for the representative classes.

Despite the relatively modest sample size of 487 patients for AI-based endeavors, we demonstrated the capability of a convolutional neural network (CNN) to distinguish prevalent craniosynostosis conditions from positional deformities or healthy toddlers.

Pertaining to the first research question, the classification task delineating the two groups—“surgery vs. no surgery indication”—yielded an accuracy of 90%, a sensitivity of 94%, and a specificity of 85%. In addressing the second research question, the accuracy metrics are encouraging, spanning a range of 84–96% for the classification across all classes, alongside appreciable sensitivity and specificity for well-represented subgroups. However, the sensitivity for the underrepresented healthy patients merely stands at 45%, which is considered inadequate.

The variations in detection performance could stem from several factors. Firstly, the limited dataset of 487 patients may not fully capture the diverse spectrum of craniofacial deformities, potentially leading to suboptimal performance, especially for underrepresented groups like healthy patients. The imbalance in the dataset, with a higher prevalence of certain deformities, might contribute to the disparity in sensitivity and specificity across different classes. Additionally, the reliance on pre-trained neural networks, particularly the ResNet152 architecture, could introduce biases. While pre-trained networks expedite learning by recognizing basic features, they might not be optimally tuned for the nuances of craniofacial morphology. Due to the scarcity of pre-trained networks for 3D data, we see another potential limitation in adapting CNNs to 2D images derived from 3D scans, as 3D information might be lost in the process.

Furthermore, the intricate nature of craniofacial anatomy and the challenge of accurately differentiating subtle deformities from normal variations may contribute to variations in performance.

A validation study consecutive to performance optimization would be needed to define a common threshold for a possible diagnostic tool. Sensitivity towards underrepresented healthy cases, marked at 45%, underscores the necessity for additional data to achieve a more robust classification. Our focus on isolated, non-syndromic craniosynostosis subtypes resulted in the exclusion of coronal or lambdoid synostoses, affecting the generalizability of findings. Furthermore, the study’s population, largely comprising patients with suspected positional deformities, introduces a potential bias in the healthy control group. To enhance the clinical relevance and address these limitations, future research should prioritize larger and more diverse datasets, consider a wider spectrum of craniosynostosis subtypes, and meticulously balance patient groups to ensure representative outcomes.

The male-to-female ratio within our cohort stands at 2.7 to 1. Notably, the existing literature cites a ratio range of 3 to 3.5 to 1 in cases of trigonocephaly and scaphocephaly, thus rendering our cohort fairly representative. However, a certain degree of bias is inherent, given that toddlers evaluated in a specialized craniofacial tertiary care center seldom exhibit symmetrical facial features (considered healthy). Our cohort also encompasses individuals who were initially suspected of positional deformity yet were subsequently categorized as healthy owing to a low cranial vault asymmetry index (CVAI). Consequently, it remains plausible that patients exhibiting mild cranial deformity were included within the healthy control group.

One of the greatest weaknesses of our study was the low number of healthy control group patients and the possible biases of this group. Acquiring 3D scans of healthy children’s head shapes from a craniofacial point of view could offer a wealth of data for an approach like ours. Inviting voluntary participation from parents, coupled with educational sessions at pediatric clinics, could facilitate this acquisition. It is imperative to obtain broad informed consent, ensuring parents fully grasp the study’s scope. Anonymizing data would uphold privacy, while a multi-center approach, involving collaboration with various healthcare centers, would yield a diverse dataset. Integrating 3D scanning within routine pediatric screenings could minimize inconvenience, and ensuring easy accessibility to scanning facilities will help avoid selection bias. Adequate training for personnel conducting the scans, robust ethical oversight, and engaging with ethical review boards will be key in maintaining quality and ethical standards. A feedback loop with participating families and healthcare providers could help continuously refine the acquisition process, making it a more streamlined and ethically sound endeavor. In any data science-based approach, the scarcity of quality data is limiting. It is therefore recommended to prepare clinical data for the future in a GDPR-conforming way to allow data use and sharing.

For our collective, another possibility to augment the population of healthy controls and thus reduce class imbalance would have been the use of a web crawler to acquire photographs of healthy infants, as utilized by Agarwal et al. [33]. Generally, deep learning algorithms perform better when large datasets are used during training [34]. However, due to the low prevalence of isolated, non-syndromic craniosynostosis, it is difficult to obtain large datasets for each craniosynostosis subtype. Smaller datasets can lead to suboptimal results. As coronal or lambdoid synostoses were grossly underrepresented in our study population, these groups were excluded. To reduce class imbalance, we adjusted weights on the loss function to emphasize underrepresented groups and applied regularization techniques.

In the scope of this project, we also tested an ensemble approach, where three different network architectures (ResNet-152, ResNext 50, and GoogLeNet) were used to classify each projection. Through this, we aimed to decrease the individual classification error and obtain an overall better classification performance. However, as the performance of the ResNet-152 approach proved close to optimal, the ensemble approach did not exhibit further improvement compared to the shown results.

An approach combining 3D photogrammetry and deep learning was conducted by de Jong et al. with a precision of 99.5% [35] for differentiation between images of the most common craniosynostosis and those of healthy patients. Yet, positional deformities were not assessed in this study, and the data required extensive preprocessing before they could be subjected to analysis. Most other studies focus on isolated characteristics and use extensive preprocessing as well. Such studies quantified the cranial shape changes consecutively to corrective surgery [36] or therapy of positional plagiocephaly [37,38].

An approach that used photography as a basis to classify sagittal, coronal, and metopic synostosis used a Res-Net with fewer hidden layers. This approach was performed with an overall sensitivity of 90.6% [33], showing comparable results with similar limitations when considering our findings. As the discrimination of positional plagiocephaly and craniosynostosis can be challenging for inexperienced practitioners, we see our approach as more suitable for this target group. Nevertheless, the sensitivity and specificity of our neuronal net leave room for improvement, especially when considering the discriminatory performance of the underrepresented groups.

Several other approaches use deep learning to classify craniosynostosis, yet most of them are conducted on the basis of CT imaging [25,39]. The utilization of machine learning not only holds promise for more accurate and early diagnosis but also for understanding the underlying morphological changes associated with craniosynostosis if methods of “explainable AI” can be applied.

However, most of these studies necessitate a large dataset and extensive preprocessing, which could be a limiting factor in real-world clinical settings. The incorporation of machine learning in craniosynostosis classification underlines an evolving interdisciplinary collaboration between medical and artificial intelligence domains, aiming to enhance diagnostic precision and patient care outcomes. The comparative examination of these machine learning approaches illustrates a burgeoning field with substantial scope for future research, particularly in developing models that require minimal preprocessing and are adept at handling a diverse range of data.

## 5. Conclusions

In conclusion, our study presents a promising approach to convolutional neural networks for craniosynostosis classification based on photogrammetry scans as a screening tool for the future. The findings support the assumption that more data for subgroups will enhance the performance, and further research with larger numbers in training is needed.

- Accuracy and specificity: The classification of surgical indication demonstrated a commendable accuracy of 90%, underscoring the diagnostic prowess. Specificity at 85% emphasizes the reliability of distinguishing cases requiring surgery from those that do not.
- Performance disparities: notably, sensitivity towards underrepresented healthy cases stood at 45%, indicating a need for increased data inclusivity for more equitable model performance.
- Ensemble approach: while an ensemble approach incorporating diverse network architectures was explored, the study revealed that the ResNet-152 approach alone yielded optimal performance, providing valuable insights into model optimization.
- Future directions: to fortify our findings, future research should prioritize expanding datasets, encompassing a broader range of craniosynostosis subtypes, and refining model sensitivity for enhanced clinical applicability.

## Figures and Tables

**Table 1:** Results after tenfold cross-validation of the test dataset (n = 96) for the indication for surgery.

	No Indication for Surgery (n = 52) ^1^	Indication for Surgery (n = 44) ^2^
Accuracy	0.90 (0.03)	0.90 (0.03)
Precision	0.88 (0.04)	0.92 (0.04)
Sensitivity	0.94 (0.03)	0.85 (0.05)
Specificity	0.85 (0.06)	0.94 (0.03)

^1^ Patients without craniosynostosis and ^2^ patients with craniosynostosis. Mean and standard deviation (parenthesis) are shown; a value of 1 represents perfect prediction in all cases.

**Table 2:** Results after tenfold cross-validation of the test dataset (n = 96) for all subgroups.

	Healthy (n = 10)	Plagiocephaly Left (n = 14)	Plagiocephaly Right (n = 26)	Scaphocephaly (n = 27)	Trigonocephaly (n = 17)
Accuracy	0.93 (0.02)	0.88 (0.03)	0.84 (0.05)	0.96 (0.03)	0.93 (0.03)
Precision	0.68 (0.12)	0.69 (0.17)	0.71 (0.09)	0.92 (0.08)	0.82 (0.10)
Sensitivity	0.73 (0.13)	0.45 (0.17)	0.76 (0.15)	0.95 (0.03)	0.80 (0.14)
Specificity	0.96 (0.02)	0.96 (0.03)	0.87 (0.07)	0.96 (0.04)	0.96 (0.03)

Differentiation of the subgroups; mean and standard deviation (parenthesis) are shown. A value of 1 represents perfect prediction in all cases.

**Table 3:** Validation metrics for surgery and no surgery indication classes. Values are normalized to 100.

Indication for	True Positive	True Negative	False Positive	False Negative
Surgery	91.35%	92.05%	7.95%	8.65%
No Surgery	92.05%	91.35%	8.65%	7.95%

**Table 4:** Validation metrics for cranial form subgroup classes. Values are normalized to 100.

Diagnosis	True Positive	True Negative	False Positive	False Negative
Healthy	48.89%	95.67%	2.00%	1.44%
Plagiocephaly left	73.36%	83.44%	1.67%	5.62%
Plagiocephaly right	88.22%	92.17%	0.67%	9.11%
Scaphocephaly	96.57%	98.56%	0.44%	2.25%
Trigonocephaly	81.22%	97.11%	0.56%	6.33%

## Data Availability

The clinical data cannot be publicly supported due to the nature of identifiable pictures of patients. Under the German GDPR regulation (DSGVO (EU) 2016/679), identifiable patient data may not be given to third parties unless explicitly consented to by the patient.
